# SNORA23 inhibits HCC tumorigenesis by impairing the 2′-O-ribose methylation level of 28S rRNA

**DOI:** 10.20892/j.issn.2095-3941.2020.0343

**Published:** 2022-01-15

**Authors:** Zhiyong Liu, Yanan Pang, Yin Jia, Qin Qin, Rui Wang, Wei Li, Jie Jing, Haidong Liu, Shanrong Liu

**Affiliations:** 1 Department of Laboratory Diagnostics, Changhai Hospital, Second Military Medical University, Shanghai 200433, China; 2 Department of Gastroenterology and Hepatology, Zhongshan Hospital, Fudan University, Shanghai 200032, China; 3 Department of Gastroenterology, Changhai Hospital, Second Military Medical University, Shanghai 200433, China; 4 Shanghai Institute of Pancreatic Diseases, Shanghai 200433, China; 5 Department of Chemistry and State Key Laboratory of Molecular Engineering of Polymers, Fudan University, Shanghai 200433 China; 6 Department of General Surgery, Changzheng Hospital, Second Military Medical University, Shanghai 200433, China; 7 Shanghai Fourth People’s Hospital, Tongji University School of Medicine, Shanghai 200081, China

**Keywords:** SNORA23, ribosome biogenesis, rRNA methylation, HCC

## Abstract

**Objective:**

The dysregulation of ribosome biogenesis is associated with the progression of numerous tumors, including hepatocellular carcinoma (HCC). Small nucleolar RNAs (snoRNAs) regulate ribosome biogenesis by guiding the modification of ribosomal RNAs (rRNAs). However, the underlying mechanism of this process in HCC remains elusive.

**Methods:**

RNA immunoprecipitation and sequencing were used to analyze RNAs targeted by ribosome proteins. The biological functions of SNORA23 were examined in HCC cells and a xenograft mouse model. To elucidate the underlying mechanisms, the 2′-O-ribose methylation level of rRNAs was evaluated by qPCR, and the key proteins in the PI3K/Akt/mTOR pathway were detected using Western blot.

**Results:**

Twelve snoRNAs were found to co-exist in 4 cancer cell lines using RPS6 pull-down assays. SNORA23 was downregulated in HCC and correlated with the poor prognoses of HCC patients. SNORA23 inhibited the proliferation, migration, and invasion of HCC cells both *in vitro* and *in vivo*. We also found that SNORA23 regulated ribosome biogenesis by impairing 2′-O-ribose methylation of cytidine^4506^ of 28S rRNA. Furthermore, SNORA23, which is regulated by the PI3K/Akt/mTOR signaling pathway, significantly inhibited the phosphorylation of 4E binding protein 1. SNORA23 and rapamycin blocked the PI3K/AKT/mTOR signaling pathway and impaired HCC growth *in vivo*.

**Conclusions:**

SNORA23 exhibited antitumor effects in HCC and together with rapamycin, provided a promising therapeutic strategy for HCC treatment.

## Introduction

As one of the most common malignant tumors worldwide, liver cancer ranks high in the number of cancer-related deaths^1^. Hepatocellular carcinoma (HCC) is one of the most frequently diagnosed primary liver cancers and is characterized by high morbidity and mortality^2^. There has been an increasing trend in the incidence and mortality of HCC^3^. More than 350,000 cases of HCC were diagnosed and there were more than 310,000 deaths in the past year in China^4^. Although numerous effective therapeutic methods, such as surgical resection, chemotherapy, liver transplantation, and immunotherapy, have been used, the prognosis of HCC remains poor^5^. Therefore, it is important to characterize the complicated underlying mechanisms of HCC, and to identify effective methods involving diagnosis and the use of therapeutic targets.

The dysregulation of ribosome biogenesis has long been considered to be a hallmark of tumor enlargement and invasion, including that of HCC^6^. Enhanced ribosome biogenesis has been observed in many cancers with poor prognoses^7^. Moreover, increasing evidence also indicates that impaired ribosome biogenesis contributes to the abnormal proteomes of cancer cells^8^. There are reports linking oncogenes and antioncogenes with the regulation of ribosome biogenesis^9^. However, we still know little about the underlying mechanisms of this process. Usually, the loss of tumor suppressor genes or deregulation of key proteins in signal transduction pathways impacts ribosome biogenesis regulatory points, such as RNA polymerases^10^. For example, impaired ribosome biogenesis usually activates the tumor suppressor protein, p53, further inducing the abnormal growth and division of cancer cells^11^. Other mechanisms are also involved in impaired ribosome biogenesis, including modification of 2′-O-ribose methylation and pseudouridylation of some ribosomal RNAs (rRNAs)^12^. Studies analyzing ribosome biogenesis in breast cancer cells with enforced aggressiveness also showed incorrect modifications of rRNAs^13^. However, what contributes to ribosome biogenesis and how this process drives the malignant transformation of HCC cells remain poorly understood.

Small nucleolar RNAs (snoRNAs) are conserved small non-coding RNAs (ncRNAs), which are usually classified as C/D-box or H/ACA-box^14^. C/D-box snoRNAs guide the 2′-O-ribose methylation of targeted rRNAs and H/ACA-box snoRNAs guide pseudouridylation by identifying complementary base pairs^15^. Impaired ribosome biogenesis is a feature of cancer cells, and snoRNAs participate in the post-transcriptional modification of rRNAs. The snoRNAs have also been shown to play a vital role in the tumorigenesis of various cancers, including HCC^16^. For example, SNORD76 was shown to be overexpressed in HCC tissues and correlated with poor prognoses of HCC patients^17^. Additionally, SNORD76 promoted the invasiveness of HCC cells by regulating the epithelial-mesenchymal transition and the Wnt/β-catenin pathway. Thus, the identifications of snoRNAs related to ribosome biogenesis in HCC are important. Dysregulations of important signaling pathways have been often observed in HCC, and have also been implicated in ribosome biogenesis and the alternation of snoRNAs^18^. Moreover, the PI3K/AKT/mTOR cascade is one of the most hyperactivated signaling pathways in HCC^19^. The eukaryotic initiation factor, 4E (eIF4E) binding protein 4E binding protein 1 (4EBP1) and ribosomal protein S6 (RPS6) are two important translational regulators that can be phosphorylated or dephosphorylated by the mTOR complex 1 (mTORC1)^20^. We therefore hypothesized that important snoRNAs may be involved in the progression of HCC by regulating the PI3K/AKT/mTOR cascade, and that characterizing its mechanism may help reveal potential therapeutic targets for HCC.

Here, we performed RNA immunoprecipitation sequencing (Rip-seq) with RPS6 antibody and further validated the low expression of SNORA23 in HCC. Aberrant expression of SNORA23 was associated the poor prognoses of HCC patients. As an H/ACA box snoRNA, SNORA23 was identified as a new guide RNA for the pseudouridylation of 28S rRNA. We found SNORA23 altered the 2′-O-ribose methylation of 28S rRNA by direct binding, and found that SNORA23 significantly inhibited the phosphorylation of 4EBP1 both *in vitro* and *in vivo*, which acted synergistically with rapamycin. Together with rapamycin, our study has provided a new strategy to control HCC.

## Materials and methods

### Cell lines and culture

Human HCC cell lines (Huh7, SMMC7721, and Hep3B) were obtained from the Chinese Academy of Sciences Cell Bank (Beijing, China). Prostate cancer (DU145), colon cancer (HCT116), and pancreatic cancer (SW1990) cells were obtained from the American Tissue Culture Collection (Manassas, VA, USA). Huh7, SMMC7721, Hep3B, and SW1990 cells were cultured in Dulbecco’s Modified Eagle Medium (Gibco, Gaithersburg, MD, USA). DU145 was cultured in RPMI 1640 medium (Gibco). HCT116 was cultured in McCoy’s 5A medium (Gibco). Media were supplemented with 10% fetal bovine serum (FBS; Gibco). Cell cultures were maintained in a humidified incubator at 37 °C in 5% CO_2_.

### Clinical samples

Clinical samples of HCC and adjacent tissues were acquired from surgical HCC patients at the Department of Surgery, Changhai Hospital (Shanghai, China). The use of clinical samples in this study was approved by the Ethics Committee of the Second Military Medical University. Informed consent was obtained from each subject. Histopathological features of clinical samples were confirmed by hematoxylin and eosin staining.

### RNA immunoprecipitation (RIP) and RNA sequencing analysis

Hep3B, DU145, HCT116, and SW1990 cells were used to perform RIP assays using RPS6 antibody (ab70227; Abcam, Cambridge, UK) and the Magna RIP RNA-Binding Protein Immunoprecipitation Kit (Millipore, Burlington, MA, USA) according to the manufacturer’s instructions. The RNAs that interacted with RPS6 were isolated, purified, and used to generate cDNA libraries, which were quantified with a Qubit 2.0 fluorimeter. Sequencing was performed with an Illumina HiSeq 2500 sequencer (Beijing Biomarker Technology, Beijing, China). RIP-seq reads were aligned to the human reference genome version 19 using the TopHat algorithm. For expression analysis, densities of genes were determined by the value of reads per kb per million reads. The accession number of the RIP-seq (RPS6) data is NCBI GEO: GSE129504.

### RNA extraction and the quantitative PCR

Total RNA of cells and tissues was isolated using RNA fast 200 (Fastagen, Shanghai, China) and TRIzol reagent (Invitrogen, Carlsbad, CA, USA) according to the manufacturers’ instructions. NanoDrop1000 (Thermo Fisher Scientific, Waltham, MA, USA) was used to evaluate the concentration and purity of the total RNA. The cDNA was synthesized using the PrimeScript™ RT Master Mix kit (TaKaRa, Shiga, Japan). Quantitative RT-PCR was performed using the SYBR^®^ Premix Ex Taq™ II (TaKaRa) and the LightCycler^®^ 480 II system (Roche, Basel, Switzerland) according to the manufacturers’ instructions. Relative expression was determined using the comparative Ct method. Glyceraldehyde 3-phosphate dehydrogenase (GAPDH) was used as an endogenous control. Primer sequences are shown in **Supplementary Table S1**.

### Recombinant plasmid construction and transfection

Full-length SNORA23 and SNORA73B were amplified using the I-5™ 2× High-Fidelity Master Mix (TsingKe, Shanghai, China) and cloned into the pcDNA3.1 vector (Invitrogen) according to the manufacturer’s instructions. The 4026-5029 region of 28S rRNA containing SNORA23 complementary sequences was amplified using the I-5™ 2× High-Fidelity Master Mix (TsingKe), and cloned into the pmirGLO vector (Promega, Madison, WI, USA), and named pmirGLO-28S-WT and pmirGLO-28S-mut. HCC cells were co-transfected with these plasmids using Lipofectamine 3000 (Invitrogen) according to the manufacturer’s protocol. Primer sequences are shown in **Supplementary Table S1**.

### SNORA23 knockout by CRISPR/Cas9

The CRISPR system was obtained from Professor Yongming Wang, Fudan University, and was performed as previously described. Two specific guide RNAs were designed using CRISPOR (http://crispor.tefor.net/) and subcloned into the CRISPR vector. HCC cells were transfected with recombined epiCRISPR vector and selected with puromycin (1 µg/mL). Knockout efficiency was verified by agarose gel electrophoresis and Sanger sequencing. The verified primers and guide RNA sequences are provided in **Supplementary Table S1**.

### Immunohistochemistry

First, tissue sections were fixed in formalin for 12 h, embedded in paraffin, deparaffinized in xylene, and rehydrated in alcohol. Second, tissue sections were incubated 45 min in a steamer using protein retrieval solution (Dako, Glostrup, Denmark), followed by 3% hydrogen peroxide solution for 10 min and protein block solution (Dako) for 20 min at room temperature. Tissue sections were then incubated overnight with antibodies against Ki-67 (9027T, Cell Signaling Technology, Danvers, MA, USA) and AKT (4691S, Cell Signaling Technology) at 4 °C followed by 30 min with the biotinylated secondary antibody (Vector Laboratories, Burlington, CA, USA), and ABC reagent (Vector Laboratories) for 30 min. Finally, 3,3′-diaminobenzidene (DAB; Dako) was used to visualize the target proteins. Images of stained tissue sections were obtained using a IX73 microscope (Olympus, Tokyo, Japan).

### Dual luciferase reporter assays

To examine whether SNORA23 directly targeted 28S rRNA, we divided the recombinant plasmids, pmirGLO-28S-WT, and pmirGLO-28S-mutalong with pcDNA3.1, pcDNA3.1-SNORA23, and empty vectors into six groups, and transfected them into Huh7 cells. Luciferase activity was determined using the Dual-Luciferase Reporter Assay System (KeyGEN Biotech, Nanjing, China) according to the manufacturer’s instructions.

### Western blot analysis

Following treatment with recombinant plasmids or chemical inhibitors, GSK2141795 (HY-15965; MCE, Princeton, NJ, USA), or rapamycin (HY-10219, MCE), HCC cells were lysed with RIPA lysis buffer (NCM Biotech, Newport, RI, USA) containing 1% protease inhibitor cocktail (Sigma-Aldrich, St. Louis, MO, USA) on ice for 30 min and boiled for 5 min at 95 °C. Proteins were separated on 4%–20% SDS-polyacrylamide gels (GenScript, Piscataway, NJ, USA) and transferred to 0.2 µm polyvinylidene difluoride membranes (Perkin Elmer Life Sciences, San Jose, CA, USA). Then, the blots were blocked with 5% nonfat dry milk (BioLight BIO, Zhejiang, China) for 1 h at room temperature and incubated with primary antibodies overnight at 4 °C. The following primary antibodies were used: AKT (4691S; Cell Signaling Technology), phospho-AKT1-S473 (AP0140; ABclonal Technology, Woburn, MA, USA), RPS6 (ab70227; Abcam), phospho-RPS6-S240/244 (AP0537; ABclonal), 4EBP1 (A1248; ABclonal), phospho-4EBP1-Thr37/46 (2855S; Cell Signaling Technology), and FBL (A13490; ABclonal). Blots were incubated with horseradish peroxidase-conjugated goat anti-rabbit IgG (H + L) (AS014; ABclonal) for 1 h at room temperature. Protein bands were visualized using ImageQuant LAS4000 (GE Healthcare, Chicago, IL, USA) and normalized to GAPDH (AC001, ABclnonal).

### Cell proliferation assays

A total of 3,000 cells were seeded into 96-well plates and the cell proliferation rate was evaluated using the Cell Counting Kit-8 assay (CCK8; Dojindo Laboratories, Kumamoto, Japan) after 2 h at 37 °C. Optical density (OD) values at 450 nm were determined using a Thermo Varioskan™ LUX (Thermo Fisher Scientific) at 0, 12, 24, 36, and 48 h after seeding. The 5′-ethnyl-0-deoxyuridine (EdU) immunofluorescence staining was conducted using the EdU Kit (Thermo Fisher Scientific) according to the manufacturer’s instructions. The stained micrographs were quantified using Image J software (National Institutes of Health, Bethesda, MD, USA).

### Cell migration assays

HCC cells transfected with recombinant plasmids or siRNAs for 24 h were washed with cold phosphate-buffered saline (PBS), digested, and seeded into the upper chamber of a Transwell chamber (Corning, Corning, NY, USA); the lower chamber contained DMEM with 10% FBS. Migrated cells were fixed using 4% paraformaldehyde solution after 12–18 h and stained with 0.1% 4′,6-diamidino-2-phenylindole. Migrated cells were counted using a IX73 microscope (Olympus).

### Wound healing migration assays

At 24 h after transfection, HCC cells were scratched with a sterile 200 µL pipette tip. Scratched cells were washed 3 times with PBS and cultured with serum-free medium. The scratched areas were recorded with a IX73 microscope (Olympus) and quantified with Image J software (National Institutes of Health).

### Ribosome activity detected by O-propargyl-puromycin (OP-Puro) incorporation

Ribosome activity was determined using an OP-Puro (HY-15680; MCE) incorporation assay. OP-Puro was added to the culture medium at a final concentration of 50 µM and incubated for 1 h; the cells were then washed twice with PBS and fixed in 0.5 mL 4% paraformaldehyde on ice for 20 min. The cells were then washed with PBS and permeabilized in 200 µL of 0.1% Triton X-100 (Bio-Light, Zhuhai, China) in PBS containing 3% FBS (Bio-Light) for 20 min at room temperature. The azide-alkyne cyclo-addition was performed using the Click-iT Cell Reaction Buffer Kit (Thermo Fisher Scientific) according to the manufacturer’s instructions, and azide conjugated to Alexa Fluor 488 (Thermo Fisher Scientific) at 2.5 µM final concentration for 30 min. The cells were then washed twice with PBS and resuspended in 200 µL Hoechst 33342 (Thermo Fisher Scientific) for 30 min. The fluorescence intensity of cells was determined using a IX73 microscope (Olympus) and acquisition software (Olympus).

### Chromatin isolation by RNA purification (ChIRP)

The 3′-biotinylated SNORA23 and scramble probes were designed by RiboBio (Guangzhou, China). The ChIRP assay is depicted in **Figure 6D** and was performed as previously described. Huh7 cells (2 × 10^7^) were seeded, grown to confluence, and cross-linked with 1% glutaraldehyde for 10 min at room temperature. The cross-linking reaction was quenched with 1.25 M glycine for 5 min, lysed with lysis buffer at 4 °C, and sonicated in a Bioruptor (TenLIN, Yancheng, China). The lysate was sonicated to a chromatin smear size range of 100–500 bp and verified by agarose gel electrophoresis. The obtained chromatin was hybridized separately with the biotinylated antisense and scramble probes for 4 h at 37 °C. The biotinylated SNORA23-bound chromatin was captured by Dynabeads^®^ MyOne™ Streptavidin C1 (Invitrogen), followed by rinsing with a wash buffer. From the ChIRP chromatin, RNA was purified from the beads using TRIzol reagent (Invitrogen). The enrichment of RNA was determined for qRT-PCR analysis.

### Animal study

The animal study was approved by the Animal Ethics Committee of Animal Experiment Center, Tongji University (Approval No. TJLAC-014–028). A total of 3 × 10^6^ Huh7 cells were suspended and subcutaneously injected into the right flanks of male 4-week-old athymic BALB/c nude mice. Tumor volume was measured using the formula: X × Y^2^ × 0.5 (where X was the longest diameter and Y was the shortest diameter). When the tumor volume reached 100 mm^3^, the mice were randomly divided into 4 groups (*n* = 7). Mice in the treatment group were injected intratumorally with rapamycin (20 µg) or pcDNA3.1-SNORA23 (50 mg), as well as a combination of rapamycin (20 µg) and pcDNA3.1-SNORA23 (50 mg) dissolved in 50 µL PBS. In the control group, mice were injected intratumorally with empty pcDNA3.1 (50 mg) dissolved in 50 µL PBS. All mice in the 4 groups were injected every 3 days. Four weeks later, the mice were euthanized and protein levels of Ki67 and AKT in xenografted tumors were examined by immunohistochemistry.

### Statistical analysis

Data are presented as the mean with standard deviation (SD). Prism 6.0 (GraphPad, San Diego, CA, USA) was used to describe the results. Statistical analyses were conducted using SPSS statistical software for Windows, version 21 (SPSS, Chicago, IL, USA). Statistical analyses were performed as described in the figure legends. **P < 0.01; *P < 0.05. A value of *P* < 0.05 was considered to be statistically significant.

## Results

### SNORA23 interacts with RPS6 and is decreased by the PI3K/AKT/mTOR cascade in HCC

**Figure 1:**
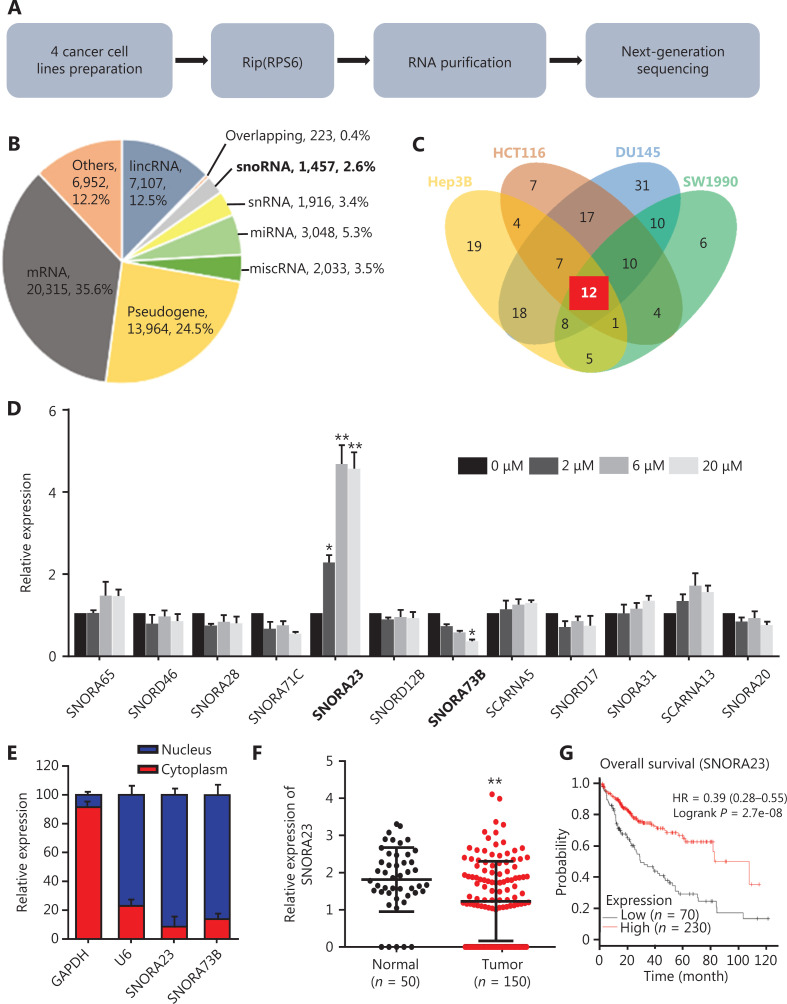
SNORA23 is affected by Akt and downregulated in hepatocellular carcinoma (HCC). (A) Schematic of the experimental methods of RPS6-RIP sequencing to identify small nucleolar RNAs involved in ribosome biogenesis. (B) The ncRNAs identified in RPS6 pull-downs. (C) Venn diagram showing 12 snoRNAs in 4 cancer cell lines from RPS6 pull-downs. (D) Related expression of the 12 snoRNAs in Huh7 cells treated with the AKT inhibitor, GSK2141795, at different concentrations. (E) Expression of SNORA23 and SNORA73B in the nucleus and cytoplasm of Huh7 cells. (F) Expression of SNORA23 was significantly downregulated in HCC samples (*n* = 150) compared with normal controls (*n* = 50) in the SNORic database. (G) The prognostic value of SNORA23 was obtained using the Kaplan-Meier Plotter online tool. Data was obtained from The Cancer Genome Atlas database. The bold text means snoRNA in RIP sequencing results (B) and significant changes of two snoRNA after GSK2141795 treatment (D). **P < 0.01; *P < 0.05. Statistical significance was determined using Student’s *t*-test.

Dysregulation of ribosome biogenesis and oncoprotein synthesis are hallmarks of numerous cancers, including HCC^21^. In addition, small nucleolar RNAs (snoRNAs) are considered to play an important role in ribosome biogenesis^22^. Moreover, the hyperactivated PI3K/AKT/mTOR cascade in HCC often promotes the phosphorylation of ribosomal protein S6 (RPS6), an indispensable translation initiation regulator in eukaryotic cells^23^. Here, we performed RNA immunoprecipitation sequencing (RIP) with RPS6 in cancer cell lines, Hep3B, HCT116, DU145, and SW1990, to identify snoRNAs involved in ribosome biogenesis (**Figure 1A**). The landscape of RNAs that interacted with RPS6 is shown in **Figure 1B** and included 20,315 messenger RNAs (mRNAs; 35.6%), 13,964 pseudogenes (24.5%), 7,107 long intervening noncoding RNA (lincRNAs; 12.5%), and 6,952 others, such as transfer RNAs (tRNAs) and rRNAs (12.2%), 3,048 microRNA (miRNAs; 5.3%), 2,033 miscellaneous RNAs (miscRNAs; 3.5%), 1,916 small nuclear RNA (snRNAs; 3.4%), 223 overlapping genes (0.4%), and 1,457 snoRNAs (2.6%) as RPS6 pulldowns in the 4 cancer cell lines. The Venn diagram indicated that only 12 snoRNAs co-existed (**Figure 1C**). These results implied that a few snoRNAs possessed critical functions in the ribosome biogenesis of various cancers.

Studies have shown that the PI3K/AKT/mTOR signaling pathway is frequently activated and involved in the progression and metastasis of HCC^24^. As a main downstream regulator, p70S6K1/2 is often phosphorylated and further regulates RPS6^25^. To clarify snoRNA-mediated regulation of ribosome biogenesis through the PI3K/AKT/mTOR-RPS6 cascade, we blocked the PI3K/AKT/mTOR signaling pathway in Huh7 cells with the AKT molecular inhibitor, GSK2141795, and detected the expression levels of 12 co-existing snoRNAs. GSK2141795 was used at 3 concentrations (2, 6, and 20 µM, and the effects were confirmed by Western blot (**Supplementary Figure S1A**). The results indicated that the expression levels of SNORA23 and SNORA73B changed significantly with increasing concentrations of GSK2141795, while the others were unchanged (**Figure 1D**). SNORA23 and SNORA73B were located within the introns of their host genes, importin 7 (*IPO7*) and the regulator of chromosome condensation (*RCC1*), respectively (**Supplementary Figure S1B**). We extracted nuclear and cytoplasmic RNAs of Huh7 cells and detected the subcellular expressions of SNORA23 and SNORA73B. More than 80% of the RNAs were expressed in the nucleus (**Figure 1E**). Next, we detected the expression levels of SNORA23 and SNORA73B in 150 HCC samples and 50 normal controls using a public database involving snoRNA in cancers (SNORic)^26^. The expression of SNORA23 was significantly downregulated in HCC samples while the expression of SNORA73B was unchanged (**Figure 1F and Supplementary Figure S1C**). We also detected their expression levels in 8 pairs of HCC tissues and adjacent non-cancerous tissues. The expression of SNORA23 was also detected in the public dataset, GSE57957. The results were consistent with the data from SNORic (**Supplementary Figure S1D–S1F**). Survival analysis indicated that the low expression level of SNORA23 was associated with poor prognoses of HCC patients (*P* = 2.7e^-08^), while SNORA73B had no relevance (*P* = 0.14) (**Figure 1G and Supplementary Figure S1G**). The survival data of HCC patients were obtained from The Cancer Genome Atlas (TCGA) dataset, which included 70 HCC patients in the SNORA23 low expression group and 230 patients in the high expression group.

These results showed that SNORA23 and SNORA73B were snoRNAs that interacted with RPS6 and were affected at least partially by the PI3K/AKT/mTOR signaling pathway. Moreover, lower expression of SNORA23 was found to correlate with poor prognoses of HCC patients, indicating its potential role in the development of HCC.

### Enforced expression of SNORA23 inhibits the proliferation, migration, and invasion of HCC cell lines

**Figure 2:**
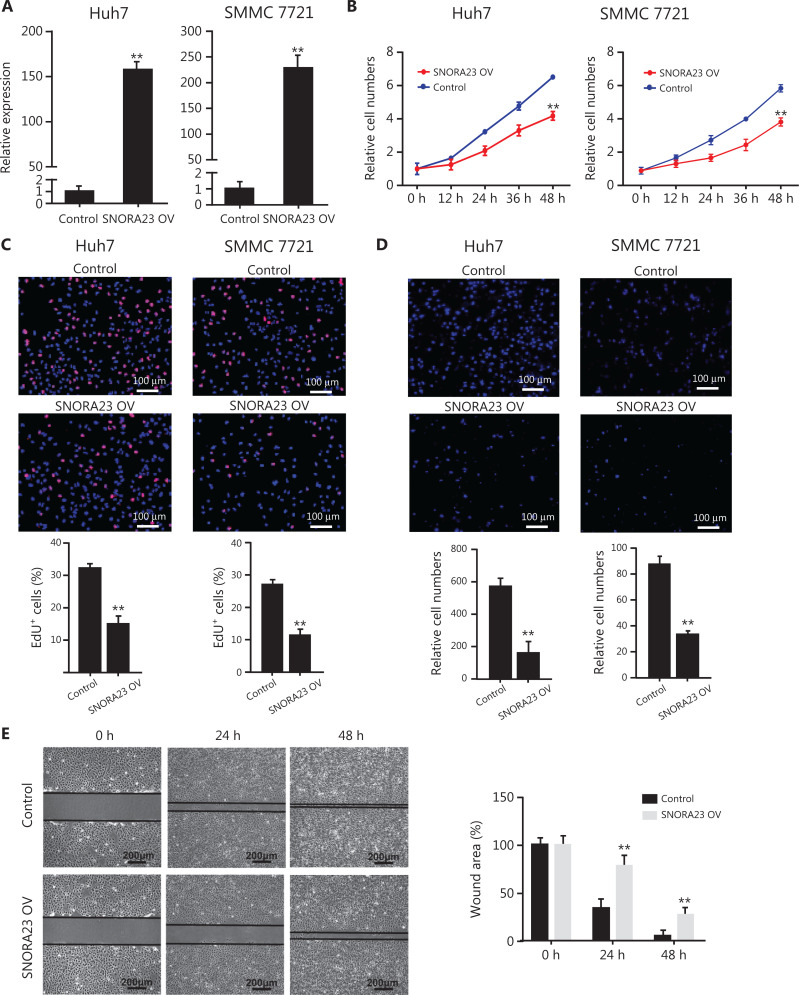
Overexpression of SNORA23 inhibited the proliferation, migration, and invasion of Huh7 and SMMC7721 cells. (A) Relative expression of SNORA23 in Huh7 and SMMC7721 cells transfected with SNORA23 overexpressed plasmids. Data are the mean ± SD (*n* = 3). (B) CCK-8 assays were performed to measure the proliferation ability of Huh7 and SMMC7721 after SNORA23 overexpression at the indicated time points. (C) The 5′-ethnyl-0-deoxyuridine assay was also performed to measure the proliferation of Huh7 and SMMC7721 cells after SNORA23 overexpression. Scale bars: 100 µm. (D) Transwell assays were performed to evaluate cell migration ability following SNORA23 overexpression in Huh7 and SMMC7721 cells. Scale bars: 100 µm. (E) Representative images of the wound healing migration assays in SNORA23-overexpressed Huh7 cells compared with controls (left panel) and the statistics of wound areas (right panel). Scale bars: 200 μm. **P < 0.01. Statistical significance was determined by the Student’s *t*-test.

Because SNORA23 was significantly decreased in HCC and low SNORA23 levels were associated with poor patient survival, we next characterized the potential effects of SNORA23 and SNORA73B in HCC cell lines. SNORA23 was overexpressed in Huh7 and SMMC 7721 cells (**Figure 2A**). The expression level of SNORA73B was not associated with HCC, but was associated with AKT. SNORA73B was also overexpressed in Huh7 cells (**Supplementary Figure S2A**). Enforced expression of SNORA23 suppressed the proliferation ability of Huh7 and SMMC 7721 cells dramatically, as evaluated by CCK-8 assays and EdU staining (**Figure 2B and 2C**). However, the enforced expression of SNORA73B failed to change the proliferation ability of Huh7 cells (**Supplementary Figure S2B and S2C**). We also assessed the migration and invasion abilities of SNORA23 and SNORA73B-overexpressing HCC cell lines by Transwell and wound healing migration assays. Transwell assays showed overexpression of SNORA23 significantly inhibited the migration and invasion abilities of Huh7 and SMMC 7721 cells nearly 3-fold (**Figure 2D**). Wound healing assays also showed the same results (**Figure 2E**). However, overexpression of SNORA73B did not change the migration and invasion ability of Huh7 cells (**Supplementary Figure S2D and S2E**). Furthermore, the overexpression of SNORA23 and SNORA73B altered expression of their host genes, *IPO7* and *RCC1*, respectively (**Supplementary Figure S2F**).

### Knockout of the SNORA23 gene by CRISPR/Cas9 promotes the proliferation, migration, and invasion of HCC cell lines

**Figure 3:**
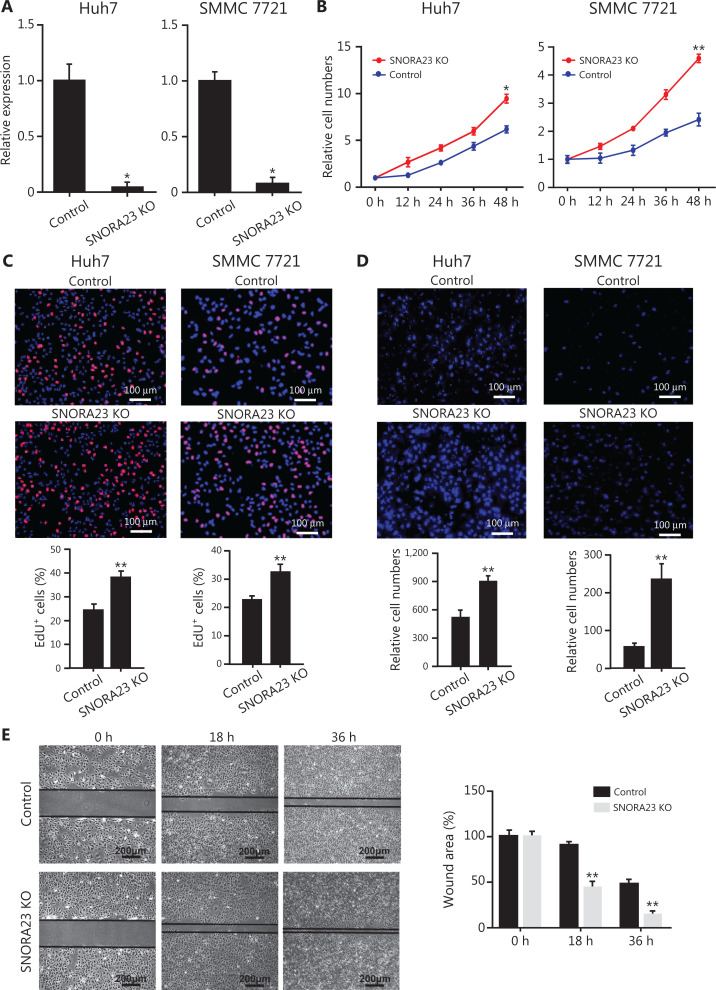
SNORA23 knockout promoted the proliferation, migration, and invasion of Huh7 and SMMC7721 cells. (A) Relative expression of SNORA23 in Huh7 and SMMC7721 cells following knockout of the *SNORA23* gene. Data are the means ± SD (*n* = 3). (B) CCK-8 assays were performed to measure the proliferation of Huh7 and SMMC7721 cells after SNORA23 knockout at the indicated time points. (C) The EdU assays were also performed to measure the proliferation of Huh7 and SMMC7721 cells after knockout of SNORA23. Scale bars: 100 µm. (D) Transwell assays were performed to evaluate cell migration ability following SNORA23 knockout in Huh7 and SMMC7721cells. Scale bars: 100 µm. (E) Representative images of the wound healing migration assays in SNORA23 knockout Huh7 cells compared with controls (left panel) and the statistics of wound areas (right panel). Scale bars, 200 μm. KO, knockout. **P < 0.01. Statistical significance was determined using the Student’s *t*-test.

To investigate the biological function of SNORA23 in HCC cells, we knocked-out the *SNORA23* gene using CRISPR/Cas9 (**Supplementary Figure S3A**). The deletion efficiency of *SNORA23* was confirmed (**Supplementary Figure S3B and S3C**); more than 90% of the expression of SNORA23 was lost in Huh7 and SMMC 7721 cells (**Figure 3A**). CCK-8 assays and EdU staining were used to evaluate the proliferation ability of SNORA23 knockout HCC cells. Compared with the control, the proliferation ability increased significantly in SNORA23 knockout cells (**Figure 3B and 3C**). Moreover, Transwell assays showed that knockout of SNORA23 significantly promoted the metastatic behavior nearly 2-fold in Huh7 cells and 3-fold in SMMC 7721 cells (**Figure 3D**). Similarly, deletion of the SNORA23 gene also markedly increased the wound-healing efficacy of HCC cell lines (**Figure 3E**). Collectively, these *in vitro* findings indicated that SNORA23 regulated the behavior of HCC cells and might act as an antioncogene in the progression of HCC.

### SNORA23 alters the 2′-O-ribose methylation of 28S rRNA

**Figure 4:**
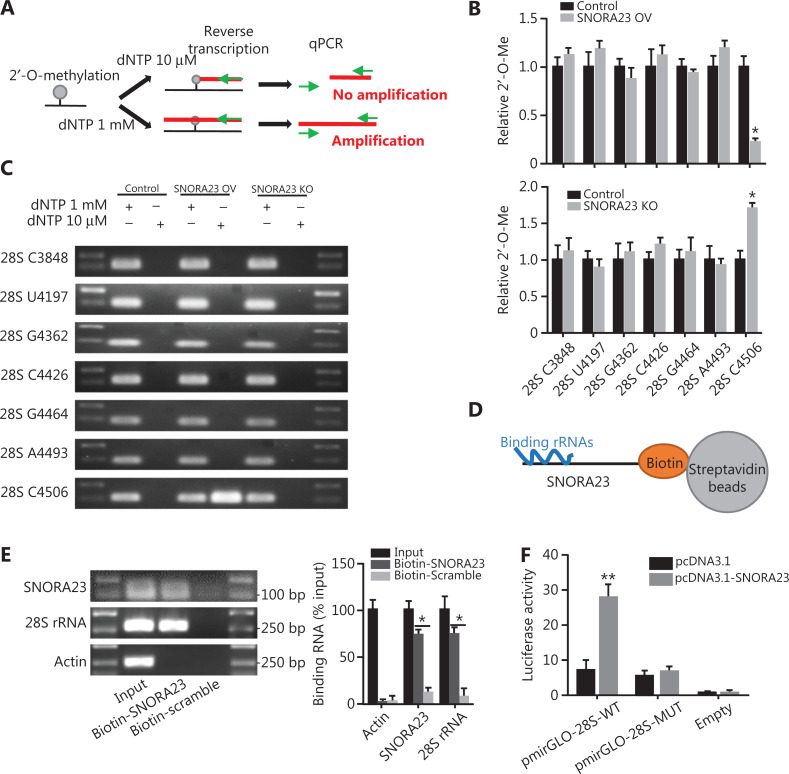
SNORA23 binds the cytidine^4506^ site of 28S rRNA and suppresses its 2′-O-ribose methylation. (A) Schematic representation of the RT-qPCR mapping assay used to measure the methylation level of rRNA. (B) The 2′-O-ribose methylation level of the common 7 sites of 28S rRNA were measured by RT-qPCR in SNORA23 overexpressed, knockout, and control Huh7 cells. (C) The results were further confirmed by gel electrophoresis. (D) Schematic diagram of the CHIRP assay. (E) Interaction between SNORA23 and 28S rRNA was detected by the CHIRP assay. (F) Luciferase activity in Huh7 cells co-transfected with SNORA23 and the indicated expression plasmids. **P < 0.01; *P < 0.05. Statistical significance was determined using the Student’s *t*-test.

As a H/ACA box snoRNA, SNORA23 has been reported to be involved in the post-transcriptional modification of 28S rRNA by guiding its pseudouridylation^27^. This has been clearly documented in the snOPY database^28^. However, it has also been reported that some H/ACA box snoRNAs also regulate the enhanced post-transcriptional methylation of specific sites located in 28S rRNA and other rRNAs expressed by HCC cells with enhanced tumor properties^29^. We next investigated if SNORA23 altered the 2′-O-ribose methylation of 28S rRNA or other rRNAs. Here, a site-specific semi-quantitative RT-qPCR based method was used (**Figure 4A**). Compared with the control, the 2′-O-ribose methylation level of cytidine^4506^ of 28S rRNA was strongly decreased in overexpressing SNORA23 and increased in SNORA23 knockout Huh7 cells (**Figure 4B and 4C**). However, no changes were observed in other known 2′-O-ribose methylation sites of 28S rRNA and 18S rRNA (**Figure 4B and 4C**, **Supplementary Figure S4A and S4B**). The 2′-O-methylation is exclusively regulated by fibrillarin (FBL), an indispensable methyltransferase in the processing of pre-rRNAs. We therefore determined if SNORA23 affected the expression of FBL. The expression of FBL was not associated with levels of SNORA23 (**Supplementary Figure S4C**), indicating that SNORA23 might impair the 2′-O-methylation of 28S rRNA cytidine^4506^ in other ways.

Accordingly, we further found that there were several nucleotide sequences between 4,480 nt to 4,740 nt of 28S rRNA, which were complementary to SNORA23 (**Supplementary Figure S4D**). This indicated that SNORA23 might directly regulate the 2′-O-methylation of 28S rRNA *via* complementary sequences. To obtain experimental evidence that SNORA23 directly bound 28S rRNA, chromatin isolation by RNA purification (CHIRP) was performed in HCC cells (**Figure 4D**). Affinity pulldown of biotin-labelled SNORA23 included endogenous 28S rRNAs (**Figure 4E**), while 5S rRNA, 5.8S rRNA, and 18S rRNA were not included (**Supplementary Figure S4E**). To further determine whether the interaction region was located in complementary sequences, we cloned the pmirGLO-28S-wild type and pmirGLO-28S-mutant plasmids to knock-out the complementary sequence of 28S rRNA. Dual luciferase reporter systems were used to evaluate interactions, and the results showed that overexpressing SNORA23 increased the luciferase activity of pmirGLO-28S-WT (**Figure 4F**). Collectively, these results showed that SNORA23 directly bound 28S rRNA and affected the 2′-O-methylation of 28S rRNA cytidine^4506^ in HCC cells.

### SNORA23 inhibits the phosphorylation of 4EBP1 and further alters ribosome translation capacity

**Figure 5:**
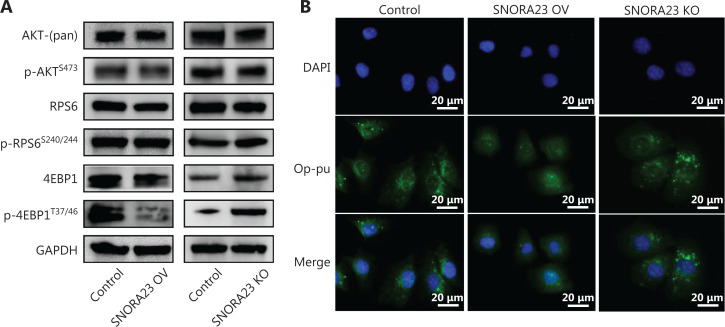
SNORA23 impairs ribosome biogenesis by affecting 4EBP1 phosphorylation. (A) The phosphorylation level of 4EBP1 was inhibited in SNORA23-overexpressing and promoted in SNORA23 knockout Huh7 cells. (B) The ribosomal biological activity was measured by O-oropargyl-puromycin staining in SNORA23-overexpressing, SNORA23 knockout, and control Huh7 cells. Scale bars: 20 µm.

SNORA23 is directly affected by the PI3K/AKT/mTOR cascade and acts as a tumor suppressor in HCC; thus, we next aimed to determine the effect of SNORA23 on downstream regulators of PI3K/AKT/mTOR signaling by using Western blot. The total expression and phosphorylation levels of AKT, RPS6, and 4EBP1 were investigated in SNORA23-overexpressing and knockout cells. Our results showed that the expression and phosphorylation levels of 4EBP1 changed significantly with changes in SNORA23 (**Figure 5A**). SNORA23 inhibited the expression and phosphorylation of 4EBP1, indicating that SNORA23 was a critical regulator of protein translation, acting mainly by targeting 4EBP1. Although SNORA23 directly bound RPS6, SNORA23 did not affect the expression and phosphorylation levels of RPS6. To evaluate the potential of SNORA23 to alter the ribosome translation capacity, we further analyzed ribosome activity using the OP-Puro incorporation assay. Compared with the control, fluorescence intensity was globally reduced in SNORA23-overexpressing cells and increased in SNORA23-deleted cells (**Figure 5B and Supplementary Figure S5A**). These results suggested that SNORA23 inhibited the phosphorylation of 4EBP1 and further altered ribosome translation capacity (**Supplementary Figure S5B**). As an inhibitor of mTORC1, rapamycin and its analogues have been shown to be useful anti-cancer agents in various tumors. However, they only suppressed the phosphorylation of RPS6. SNORA23 may therefore inhibit EBP1 and act synergistically with rapamycin, targeting the PI3K/AKT/mTOR-RPS6/4EBP1 cascade.

### Combined treatment with rapamycin and SNORA23 inhibits the PI3K/AKT/mTOR-RPS6/4EBP1 cascade and HCC growth *in vivo*

**Figure 6:**
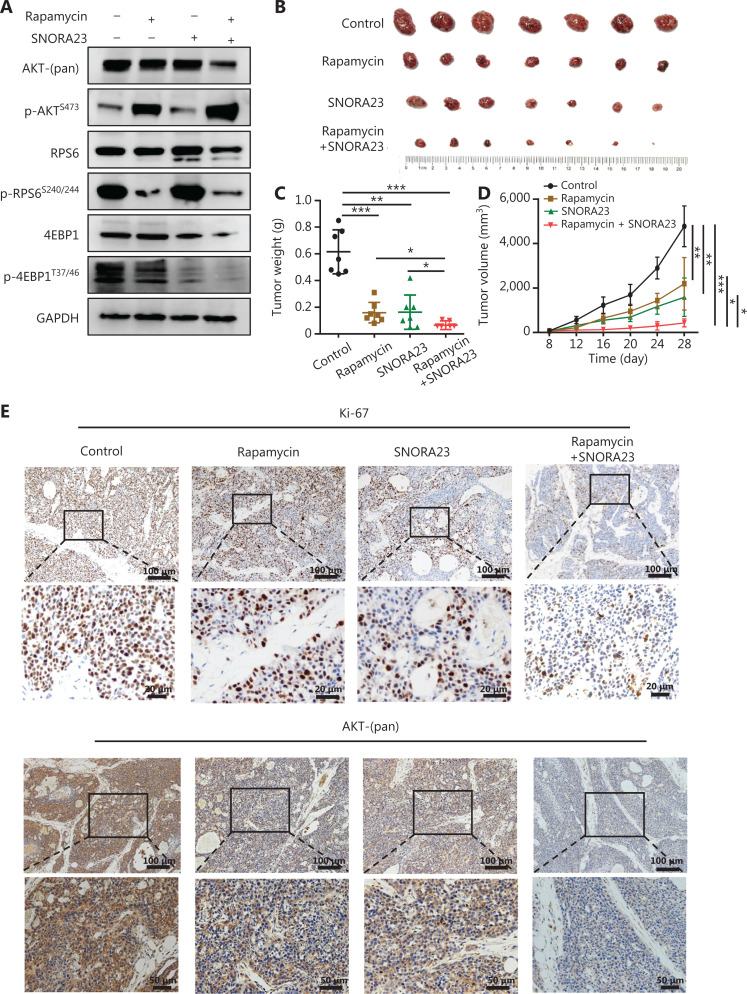
Combined treatment with rapamycin and SNORA23 blocked the PI3K/AKT/mTOR-RPS6/4EBP1 cascade and inhibited hepatocellular carcinoma tumor growth *in vivo.* (A) Western blot analysis of the total protein expression levels and phosphorylation levels in Huh7 cells treated with rapamycin and SNORA23. (B) Tumors formed from the Huh7 cells treated with rapamycin, SNORA23, rapamycin+SNORA23, and PBS as control in nude mice are shown (*n* = 7). (C) Xenograft tumor weights in the control, rapamycin, SNORA23, and rapamycin+SNORA23 groups. Data are the means ± SD (*n* = 7) (D) Xenograft tumor growth rates in the control, rapamycin, SNORA23, and rapamycin+SNORA23 groups. Data are the means ± SD (*n* = 7) (E) Immunohistochemistry analysis of Ki-67 (upper) and AKT (bottom) protein expression levels in xenograft tumors in control, rapamycin, SNORA23, and rapamycin+SNORA23 groups. Scale bars: 100 µm, 20 µm, and 50 µm. ***P < 0.001; **P < 0.01; *P < 0.05. Statistical significance was determined using the Student’s *t*-test.

As a downstream effector of the PI3K/AKT signaling pathway, mTORC1 plays a critical role in the progression of HCC *via* regulation of RPS6 and 4EBP1^20^. Rapamycin is an inhibitor of mTORC1 and effectively inhibits the phosphorylation of RPS6, but not 4EBP1 (**Supplementary Figure S6A**). We found that SNORA23 inhibited the phosphorylation of 4EBP1 *in vitro*. Thus, we further aimed to determine if SNORA23 also inhibited the phosphorylation of 4EBP1 and acted in combination with rapamycin to inhibit the PI3K/AKT/mTOR-RPS6/4EBP1 cascade. We determined the expression and phosphorylation levels of AKT, RPS6, and 4EBP1 in rapamycin-treated, SNORA23-overexpressing, and SNORA23-overexpressing rapamycin-treated Huh7 cells. Rapamycin and SNORA23 impaired the phosphorylation of 4EBP1 and RPS6, respectively (**Figure 6A**), while treatment of SNORA23 overexpressing cells with rapamycin inhibited the total protein levels of AKT and increased the phosphorylation level of AKT (**Figure 6A**).

We also generated transplant mouse models by subcutaneously injecting HCC cells. PBS (control), rapamycin, SNORA23, and a mixture of SNORA23 and rapamycin solutions were injected into 4 groups of transplant tumors intermittently when the xenografts grew to a suitable size. The transplant tumors were small; the tumor weight and growth rate declined in groups treated with rapamycin and SNORA23, and were significantly suppressed in the group treated with both (**Figure 6B–6D and Supplementary Figure S6B**). Immunohistochemistry showed that the Ki-67 and AKT staining of xenografts was reduced in the SNORA23 and rapamycin combined treatment group compared with the control group (**Figure 6E**). These results showed that treatment with SNORA23 and rapamycin in combination effectively inhibited the PI3K/AKT/mTOR-RPS6/4EBP1 cascade and the tumorigenesis of HCC both *in vitro* and *in vivo*, providing a promising therapeutic strategy for treatment of HCC.

## Discussion

**Figure 7:**
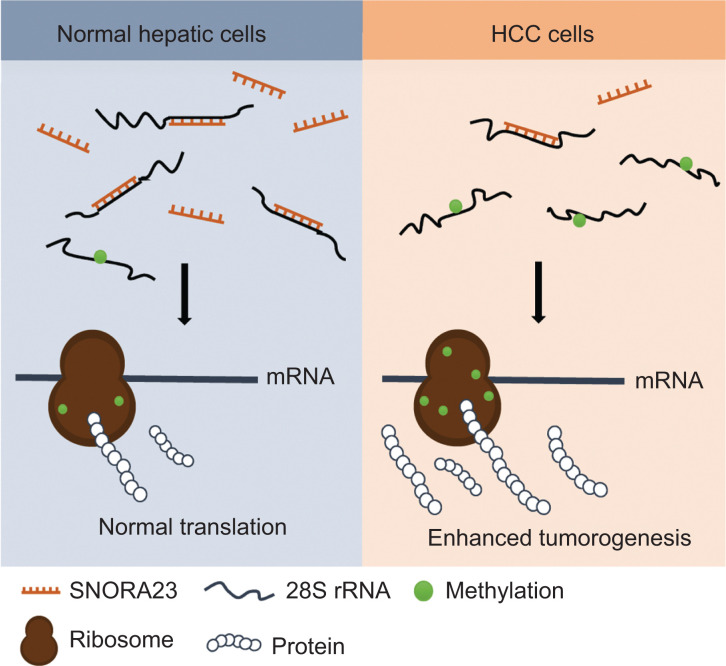
Proposed model for SNORA23 regulation of ribosome biogenesis by impairing 28S rRNA methylation and further inhibition of hepatocellular carcinoma progression.

For the first time, we report SNORA23 regulation of ribosome biogenesis by impairing methylation of 28S rRNA, resulting in inhibition of the progression of HCC by targeting PI3K/Akt/mTOR signaling. Expression of SNORA23 was low in both HCC tumor tissues and cell lines, and correlated with poor prognoses of HCC patients. We showed that SNORA23 inhibited the proliferation, migration, and invasion of HCC cells *in vitro* and *in vivo*, indicating that SNORA23 functioned as a tumor inhibitor in HCC. Moreover, SNORA23 significantly inhibited the phosphorylation of 4EBP1 and together with rapamycin, provided a promising therapeutic strategy for HCC (**Figure 7**).

Ribosome biogenesis is complicated and plays an essential role in the formation of the proteome^30^. Numerous rRNAs and ribosomal proteins have been identified in this process, especially in cancer cells^31^. Recently, an increasing number of studies have indicated that the dysregulation of ribosome biogenesis might affect the translation capacity and cellular processes such as proliferation, apoptosis, DNA damage, and stem cell self-renewal of cancer cells^7^. For example, inhibition of 18S rRNA and 28S rRNA expressions caused defective ribosome synthesis and induced accumulation of p53^32^. Moreover, studies have demonstrated that rRNAs catalyze and control protein synthesis through their ribozyme activity, which can be regulated by posttranscriptional modifications including methylation and pseudouridylation^33^. To investigate the mechanism of ribosome synthesis in HCC, we performed RIP-seq with RPS6 in cancer cell lines and determined global transcriptome binding with RPS6. After further analysis, we determined that the expressions of SNORNA23 and SNORA73B were regulated by the AKT molecular inhibitor, GSK2141795, indicating they may be related to the PI3K/Akt/mTOR signaling. Only the expression of SNORA23 was inhibited in HCC, indicating the tumor suppressor role of SNORA23.

The host genes of *SNORA23* and *SNORA73B* are *IPO7* and *RCC1*, respectively. As an H/ACA box snoRNA, the *SNORA23* gene is located between exons 13 and 14 of the *IPO7* gene. We found that overexpression of SNORA23 and SNORA73B inhibited expression of their host genes. IPO7 encodes a protein that participates in nuclear import and also directly binds nuclear pore complexes, where it competes for binding sites with importin-beta and transportin. The functions of RCC1 include accelerating the cell cycle and DNA repair, and inhibiting DNA damage-induced cell senescence. No studies of the biological functions of IPO7 and RCC1 in HCC were found. Whether SNORA23 displays antitumor effects through its host gene is largely unknown and will required further investigation. A previous study showed that SNORA23 is upregulated in pancreatic ductal adenocarcinoma (PDAC) tissues and cell lines. Knockout of SNORA23 inhibited the expression of its downstream effector SYNE2, which was associated with the proliferation and invasion of PDAC cells^34^. In our study, we identified SNORA23 as a downstream factor of PI3K/AKT/mTOR signaling and showed that SNORA23 played a tumor suppressor role in HCC. Overexpression of SNORA23 inhibited the proliferation, invasion, and metastasis of HCC cells and knockout of SNORA23 using CRISPR/Cas9-promoted malignant transformation of HCC cells. Furthermore, SNORA23 impaired ribosome translational initiation by suppressing phosphorylation of 4EBP1. These results indicated that SNORA23 was a key regulator of ribosome biogenesis and inhibited the carcinogenesis of HCC.

Recently, it has been shown that altering rRNA methylation through modulation of FBL expression affected the regulation of translation^35^. FBL overexpression was shown to directly result in the tumorigenesis of normal cells by altering the transcription and translation of key oncogenes^36^. The decreased levels of several rRNAs in human cells have been associated with a decrease in translation fidelity^37^. In acute myelogenous leukemia, global loss of C/D box snoRNAs represses the 2′-O-ribose methylation level of rRNAs and inhibits the self-renewal potential of leukemia cells^38^. In this study, we found that SNORA23 impaired the 2′-O-ribose methylation level of 28S rRNA cytidine^4506^, but had no effect on the expression of FBL. RNA pull-down and dual luciferase assays also confirmed that SNORA23 directly bound to 28S rRNA.

Improved knowledge of oncogenes and signaling pathways that participate in the proliferation, differentiation, angiogenesis, invasion, and metastasis of tumor cells has led to the identification of several possible therapeutic targets^39^. These also promoted the development of targeted therapies for HCC patients^40^. As an allosteric partial inhibitor of mTORC1, the anti-cancer effects of rapamycin *via* inhibition of mTORC1 have been reported in several human tumors^41^. Rapamycin only suppresses the phosphorylation of RPS6 and does not affect 4EBP1 phosphorylation, showing limiting clinical therapy efficacy^42^. However, we found that combining rapamycin with SNORA23 significantly inhibited AKT expression and hepatocarcinogenesis by blocking RPS6 and 4EBP1 phosphorylation, both *in vitro* and *in vivo*.

## Conclusions

In summary, we showed that SNORA23, which was regulated by the PI3K/AKT/mTOR signaling pathway, played a critical role in ribosome biogenesis and HCC tumorigenesis. SNORA23 directly bound to 28S rRNA and impaired the methylation of a specific site. In addition, the combination of SNORA23 and rapamycin blocked two main cascades of PI3K/AKT/mTOR signaling, RPS6 and 4EBP1, resulting in decreased AKT levels and tumorigenesis in an HCC mouse model. These results showed a possible innovative therapy for HCC.
