# Supplementary material for: SNORA23 inhibits HCC tumorigenesis by impairing the 2′-O-ribose methylation level of 28S rRNA
Source: Cancer Biol Med. 2022 Jan 15;19(1):104–19. doi: 10.20892/j.issn.2095-3941.2020.0343 (PMC8763008; doi:10.20892/j.issn.2095-3941.2020.0343)
Supplement: Supplementary file 1 [file cbm-19-104-s001.pdf]

# Supplementary materials

## Materials and methods

### Quantification of rRNA methylation by RT-qPCR

Site-specific ribosome RNA methylation levels were measured as previously reported with minor modification[1]. As shown in **Figure 4A**, reverse transcription was performed using 500 ng of total RNA in the presence of 200 units of RNase H minus M-MLV reverse transcriptase (Promega), 40 units of RNasin Ribonuclease Inhibitor (Promega), 1 μM of each reverse primer targeting a sequence downstream of a specific methylation site, and 10 μM or 1 mM dNTPs (TaKaRa). Reactions were incubated at 37 °C for 5 min, and then stopped by incubation at 70 °C for 15 min. Quantitative amplification of the targeted cDNAs was assessed by real-time PCR using a SYBR® Premix Ex Taq™ II (TaKaRa) and confirmed by agarose gel electrophoresis. The methylation ratio was calculated following the function $2^{(CT\ low - CT\ high)}$. The primers are shown in **Supplementary Table S1**.

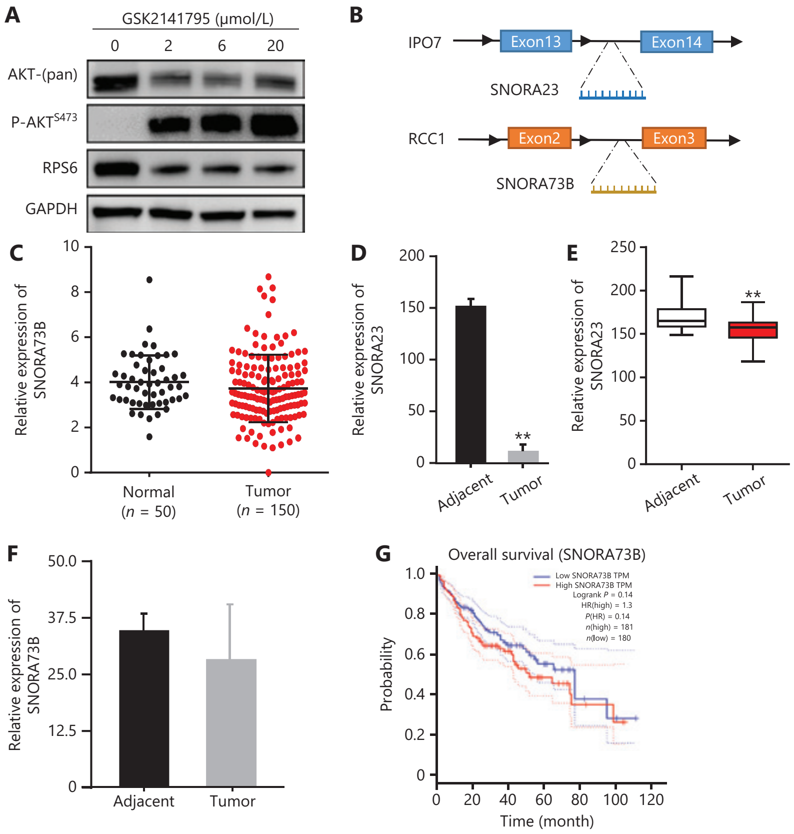


**Figure S1** The expression level of SNORA73B was unchanged in hepatocellular carcinoma. (A) Total protein and phosphorylation levels of AKT and RPS6 changed after treatment of Huh7 cells with GSK2141795. (B) SNORA23 and SNORA73B loci are shown in their host genes as IPO7 and RCC1, respectively. (C) Expression of SNORA73B was unchanged in HCC samples ($n$ = 150) compared with normal controls ($n$ = 50) in the SNORic database. (D) Relative expression of SNORA23 in HCC tissues compared with corresponding adjacent tissues ($n$ = 8). (E) Relative expression of SNORA23 in HCC tissues compared with corresponding adjacent tissues (GSE57957, $n$ = 39). (F) Relative expression of SNORA73B in HCC tissues compared with corresponding adjacent tissues ($n$ = 8). (G) Prognostic values of SNORA73B were obtained using the gene expression profiling interactive analysis (GEPIA) online tool (no data was found using the Kaplan-Meier Plotter online tool). $^{**}P < 0.01$. Statistical significance was determined using Student's $t$-test.

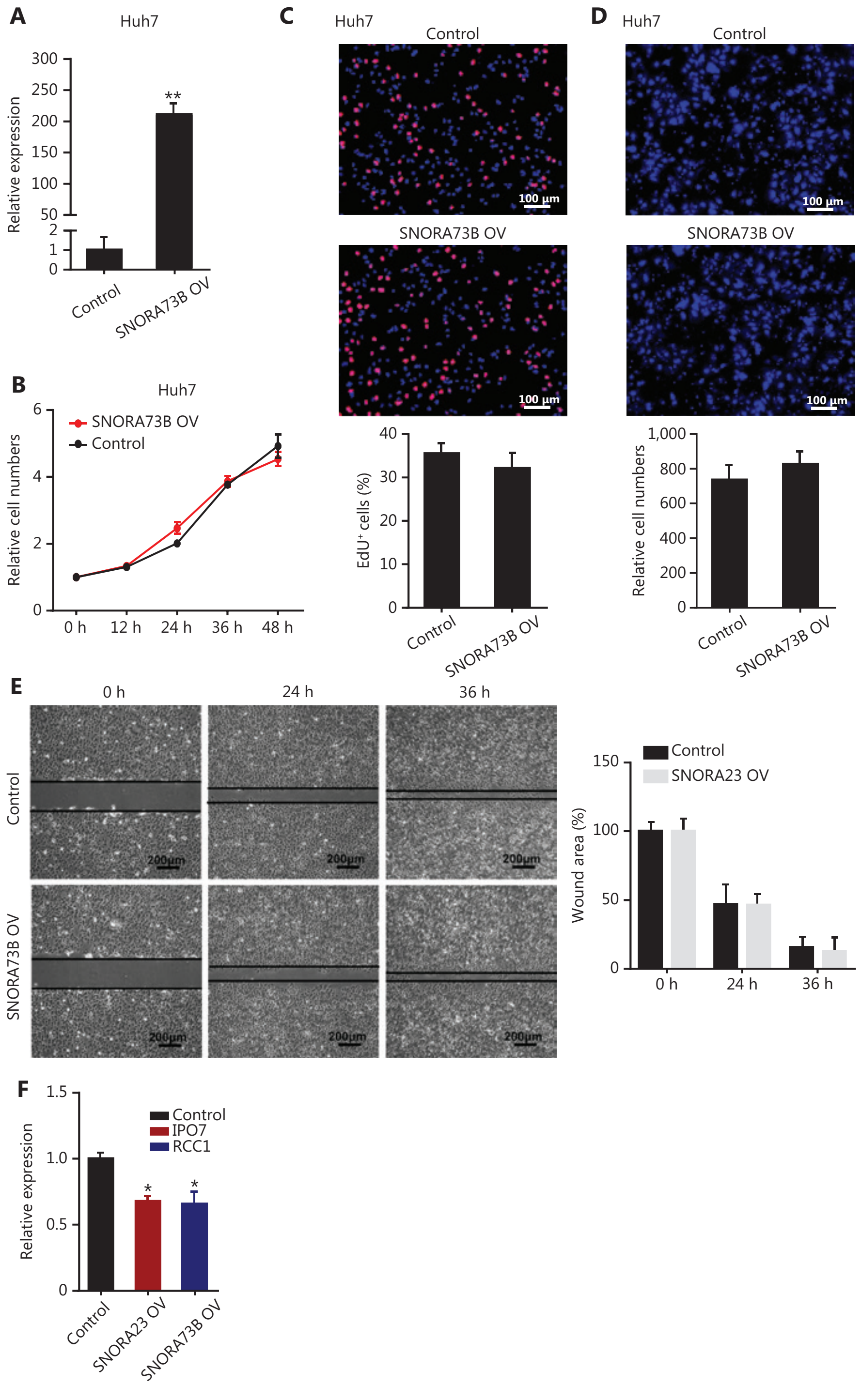


**Figure S2** Overexpression of SNORA73B had no effect on the behavior of Huh7 cells. (A) SNORA73B was overexpressed in Huh7 cells. Data are the mean ± SD ($n$ = 3). (B) CCK8 assays were performed to assess proliferation ability of SNORA73B-overexpressing Huh7 cells at the indicated time points. (C) The 5′-ethynyl-0-deoxyuridine assays were also performed to assess proliferation of SNORA73B-overexpressing Huh7 cells. Scale bars: 100 μm. (D) Transwell assays were performed to evaluate the cell migration ability of SNORA73B-overexpressing Huh7 cells. Scale bars: 100 μm. (E) Representative images of the wound healing migration assays in SNORA73B-overexpressing Huh7 cells compared with controls (left panel) and the statistical analyses of wound areas (right panel). Scale bars: 200 μm. (F) Relative expression levels of IPO7 and RCC1 in SNORA23 and SNORA73B-overexpressing Huh7 cells, respectively. **$P$ < 0.01; *$P$ < 0.05. Statistical significance was determined using the Student's $t$-test.

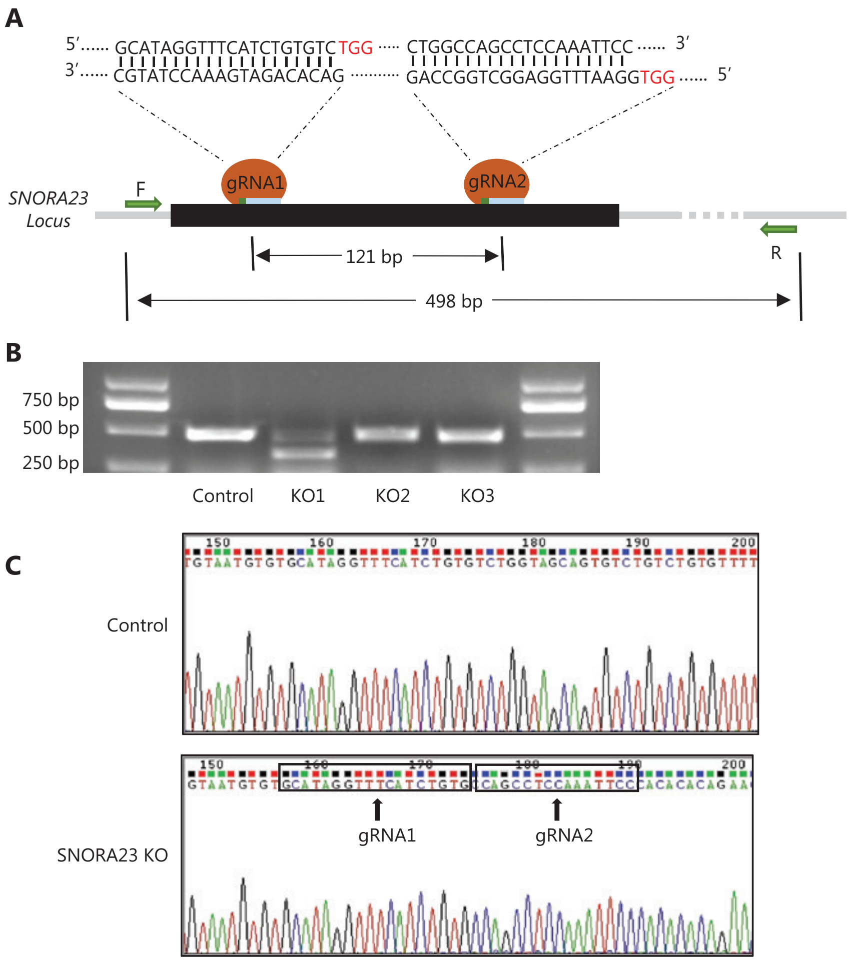


**Figure S3** The SNORA23 knockout mediated by the CRISPR/Cas9 system. (A) Schematic diagram shows two guide RNA sequences and PCR primers used to examine deletion efficiency. (B) Compared with the control, northern blot analysis indicated that KO1 screened from several single clones significantly knocked-out the *SNORA23* gene. (C) The sequencing results of KO1.

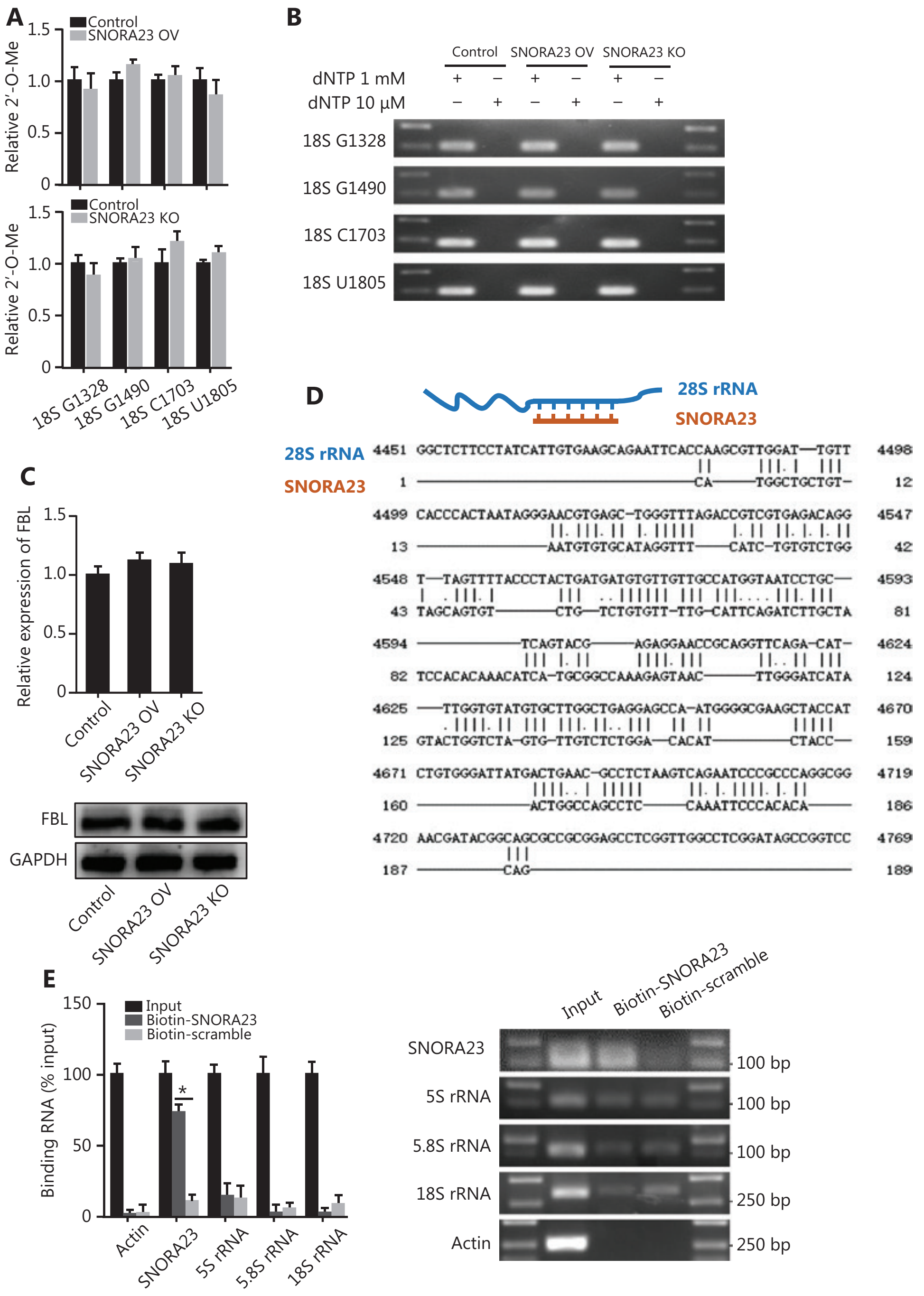


**Figure S4** SNORA23 has no effect on the 2′-O-ribose methylation of 18S rRNA. (A) The 2′-O-ribose methylation levels of the 4 common sites of 18S rRNA were measured by RT-qPCR in SNORA23-overexpressing, knockout, and control Huh7 cells. (B) Gel electrophoresis results. (C) The mRNA and protein expression levels of fibrillarin (FBL) were measured in SNORA23-overexpressing, knockout, and control Huh7 cells. (D) The predicted binding region for SNORA23 in 28S rRNA. (E) Interaction of SNORA23 and 5S, 5.8S, and 18S rRNA were detected by CHIRP assays. Data is shown as the mean ± SD. *$P$ < 0.05. Statistical significance was determined using the Student's $t$-test.

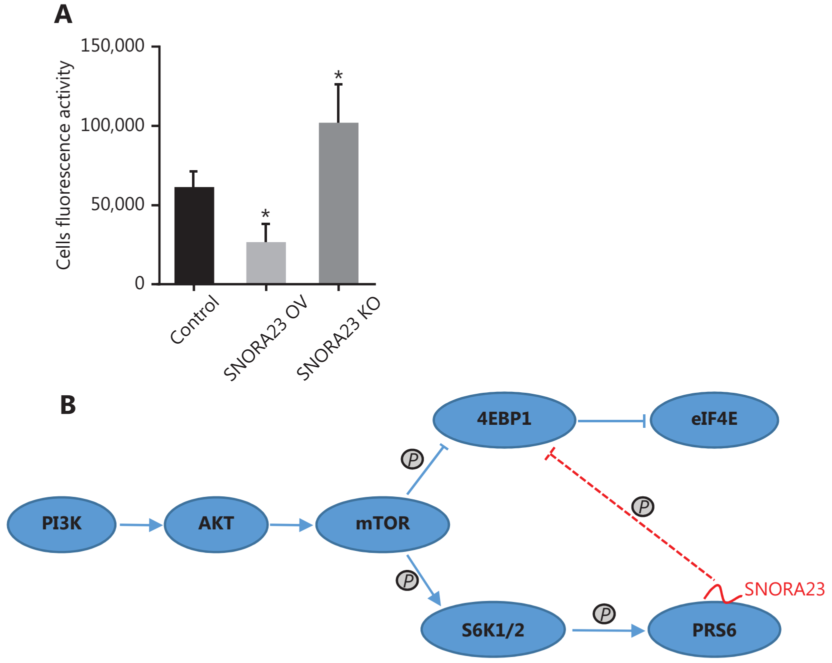


**Figure S5** SNORA23 inhibits the phosphorylation of 4EBP1. (A) The fluorescence of SNORA23-overexpressing, SNORA23 knockout, and control Huh7 cells stained with O-oropargyl-puromycin. Data are expressed as the mean ± SD. (B) The PI3K/Akt/Mtor-SNORA23 cascade. *$P$ < 0.05. Statistical significance was determined using the Student's $t$-test.

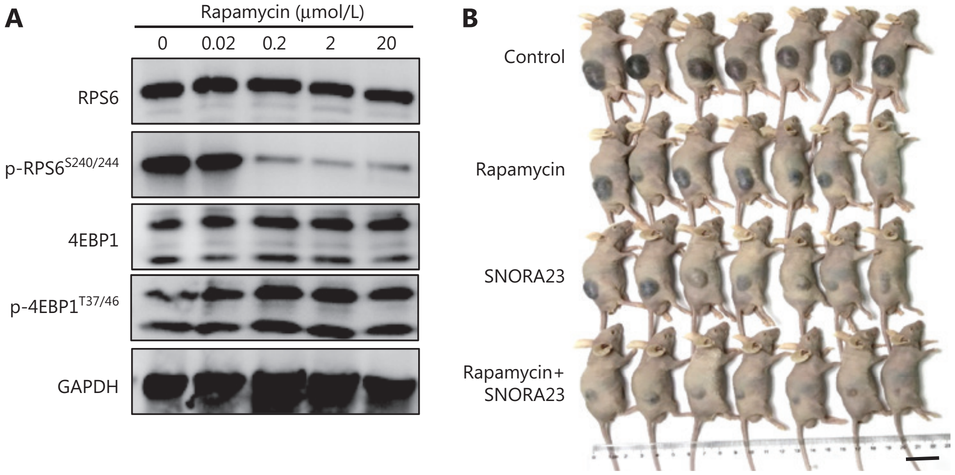


**Figure S6** Combined treatment with rapamycin and SNORA23 blocked PI3K/AKT/mTOR signaling. (A) Western blot analysis shows rapamycin significantly inhibited the phosphorylation level of RPS6 and had no effect on 4EBP1. (B) Integral images of mice xenograft tumors in control, rapamycin, SNORA23, and rapamycin+SNORA23 groups.

**Table S1** List of primers sequences used in this study

| Real-time qPCR primers sequences | Sequence (5′→3′) |
|---|---|
| SNORA65 sense | 5′-TGCAATCCAGTGGTGAGCTG-3′ |
| SNORA65 anti-sense | 5′-GTTCCCATGCTTTCGGCAC-3′ |
| SNORD46 sense | 5′-AGAATCCTTAGGCGTGGTTGT-3′ |
| SNORD46 anti-sense | 5′-ATGACAAGTCCTTGCATTGGC-3′ |
| SNORA28 sense | 5′-AAGCAACACTCTGTGGCAGAT-3′ |
| SNORA28 anti-sense | 5′-TGGGAGAGAGAACACCGGAA-3′ |
| SNORA71C sense | 5′-TGGTAGTGCAGGGAGAGGAA-3′′ |
| SNORA71C anti-sense | 5′-GGAAAGCTCCAGGGTTTGGA-3′ |
| SNORA23 sense | 5′-TGGTAGCAGTGTCTGTCTGTG-3′ |
| SNORA23 anti-sense | 5′-CCAAGTTACTCTTTGGCCGC-3′ |
| SNORD12B sense | 5′-GGTGGCCTTGCCTTCATTTC-3′ |
| SNORD12B anti-sense | 5′-GATCCAAGGTTGCGCTCAGG-3′ |
| SNORA73B sense | 5′-CTGTCCAAGTGGCATAGG-3′ |
| SNORA73B anti-sense | 5′-CCAGTCATTGTCCACAGG-3′ |
| SCARNA5 sense | 5′-TGAATGTCACGGTCCCTTTGT-3′ |
| SCARNA5 anti-sense | 5′-GCTGCTCCATGATCCCATACA-3′ |
| SNORD17 sense | 5′-GTAGCCTGAAAATGCCCTGC-3′ |
| SNORD17 anti-sense | 5′-CCCTGCTGACACCAACCATT-3′ |
| SNORA31 sense | 5′-CTGCATCCACTGATAGACCTTGA-3′ |
| SNORA31 anti-sense | 5′-AGACAGACAGAAAGCGCAGG-3′ |
| SCARNA13 sense | 5′-AGCACACCAGACTTGCAGAA-3′′ |
| SCARNA13 anti-sense | 5′-ACATGGAACAGCTGGCTCTC-3′ |
| SNORA20 sense | 5′-ACGAGCAGTTATACGCATGG-3′ |
| SNORA20 anti-sense | 5′-AGTCCCTGCAGAAGAGATGAA-3′ |
| SNORA23 KO check sense | 5′-GCAGTGAACTAGGCTACATGAG-3′ |
| SNORA23 KO check anti-sense | 5′-AAGGCTGTTTGAAGGTTCTGAT-3′ |
| IPO7 sense | 5′-ATCGAGAAACAGCACCAGGG-3′ |
| IPO7 anti-sense | 5′-CTACCAATGGACTCCGCTCC-3′ |
| RCC1 sense | 5′-CTGACCAGAAAACCCGACCA-3′ |
| RCC1 anti-sense | 5′-CCACTGATGTGTCCCTTCCC-3′ |
| FBL sense | 5′-CCAAGAAGAGGACCAACATCAT-3′ |
| FBL anti-sense | 5′-GAGGCTGTGGAGTCAATGC-3′ |
| 5S sense | 5′-TCTACGGCCATACCACCCT-3′ |

**Table S1** Continued

| Real-time qPCR primers sequences | Sequence (5′→3′) |
|---|---|
| 5S anti-sense | 5′-CTACAGCACCCGGTATTCC-3′ |
| 5.8S sense | 5′-ACTCTTAGCGGTGGATCA-3′ |
| 5.8S anti-sense | 5′-AAGTGCGTTCGAAGTGTC-3′ |
| 18S sense | 5′-ATCAGATACCGTCGTAGTTC-3′ |
| 18S anti-sense | 5′-CCAGAGTCTCGTTCGTTAT-3′ |
| 28S sense | 5′-GCGAATGATTAGAGGTCTTG-3′ |
| 28S anti-sense | 5′-GGCAGGTGAGTTGTTACA-3′ |
| 18S G1328 sense | 5′-TCTTTC TCGATTCCGTGGGTG-3′ |
| 18S G1328 anti-sense | 5′-CATGCCAGAGTCTCGTTCGT-3′ |
| 18S G1490 sense | 5′-TTCAGCCACCCGAGATTGAG-3′ |
| 18S G1490 anti-sense | 5′-CGCTGAG CCAGTCAGTGTAG-3′ |
| 18S C1703 sense | 5′-GCGTTGATTAAGTCCCTGCC-3′ |
| 18S C1703 anti-sense | 5′-GGGCCTCACTAAACCATCCA-3′ |
| 18S U1805 sense | 5′-GCGGAGC GCTGAGAAGAC-3′ |
| 18S U1805 anti-sense | 5′-GATCCTTCCGCAGGTTCAC-3′ |
| 28S C3848 sense | 5′-ACGCGCATGAATGGATGAAC-3′ |
| 28S C3848 anti-sense | 5′-GGTCTTCT TTCCCCGCTGAT-3′ |
| 28S U4197 sense | 5′-TCAAACGGTAACGCAGGTGT-3′ |
| 28S U4197 anti-sense | 5′-GATCAAGCGAGCTTTTGCCC-3′ |
| 28S G4362 sense | 5′-TGGGTTTT AAGCAGGAGGTGT-3′ |
| 28S G4362 anti-sense | 5′-GACGTCGCTATGAACGCTTG-3′ |
| 28S C4426 sense | 5′-ACAGGGATAACTGGCTTGTGG-3′ |
| 28S C4426 anti-sense | 5′-CAATCCA ACGCTTGGTGAAT-3′ |
| 28S G4464 sense | 5′-TGAAGCAGAATTCGCCAAGC-3′ |
| 28S G4464 anti-sense | 5′-TCACGACGGTCTAAACCCAG-3′ |
| 28S A4493 sense | 5′-AAGCAGA ATTCACCAAGCGT-3′ |
| 28S A4493 anti-sense | 5′-CTCACGACGGTCTAAACCCA-3′ |
| 28S C4506 sense | 5′-GCGTTGGATTGTTCACCCAC-3′ |
| 28S C4506 anti-sense | 5′-ACCATGGC AACAACACATCA-3′ |
| U6 sense | 5′-CCTTCGGGGACATCCGATAA-3′ |
| U6 anti-sense | 5′-CTCGATTTGTGCGTGTCATCC-3′ |
| GAPDH sense | 5′-GGTCTCCTCTGACTTCAACA-3′ |
| GAPDH anti-sense | 5′-GTGAGGGTCTCTCTCTTCCT-3′ |
| Actin sense | 5′-CATGTACGTTGCTATCCAGGC-3′ |
| Actin anti-sense | 5′-CTCCTTAATGTCACGCACGAT-3′ |

**Table S1** Continued

| Vectors construction primers sequences | Sequence (5′→3′) |
|---|---|
| SNORA23 OV forward | 5′-GGATCCCATGGCTGCTGTAATGTGTG-3′ |
| SNORA23 OV reverse | 5′-GAATTCCTGTGTGTGGGAATTTGGAG-3′ |
| SNORA73B OV forward | 5′-GGATCCTCCAACGTGGATACCCTGGG-3′ |
| SNORA73B OV reverse | 5′-GAATTCATATGTTTCCTGCATGGTTTG-3′ |
| SNORA23 Cas9 forward | 5′-GGACGAAACACCGAGAAGAGCGCATAGGTTTCATCTGTGTCG-3′ |
| SNORA23 Cas9 reverse | 5′-ATTTCTAGCTCTAAAACCGAAGAGCCTGGCCAGCCTCCAAATTC-3′ |
| 28S-1 forward | 5′-CTGCGGGCCGCCGGTGAAATACCAC-3′ |
| 28S-1 reverse | 5′-GTGAATTCTGCTTCACAATGATAG-3′ |
| 28S-2 forward | 5′-CGCCGCGGAGCCTCGGTTGGC-3′ |
| 28S-2 reverse | 5′-GACAAACCCTTGTGTCGAGGGCTG-3′ |

KO, knockout; OV, overexpression.

# Reference

1 Belin S, Beghin A, Solano-Gonzàlez E, Bezin L, Brunet-Manquat S, Textoris J, et al. Dysregulation of ribosome biogenesis and translational capacity is associated with tumor progression of human breast cancer cells. PLoS One. 2009; 4: e7147.
